# Dahuang Fuzi Decoction Attenuates Renal Fibrosis and Ameliorates Mitochondrial Dysfunction in Chronic Aristolochic Acid Nephropathy

**DOI:** 10.1155/2017/9536458

**Published:** 2017-03-21

**Authors:** Guang-xing Shui, Dong Sang, Xun Yin, Yun Cai, Wei Sun

**Affiliations:** ^1^The First Affiliated Hospital of First Clinical Medical School, Nanjing University of Chinese Medicine, Nanjing 210029, China; ^2^Department of Nephrology, Changshu No. 2 People's Hospital, Changshu 215500, China; ^3^Department of Second Clinical Medical School, Nanjing University of Chinese Medicine, Nanjing 210029, China

## Abstract

*Objectives*. The effects of the traditional formula Dahuang Fuzi Decoction (DFD) on chronic aristolochic acid nephropathy (AAN) in mice and its underlying mechanisms were studied.* Methods*. Mice were randomly divided into the following six groups: the control group, the model group (AAN), the saline-treated group (AAN + vehicle), the normal dose DFD-treated group (AAN + NDFD), the high dose DFD-treated group (AAN + HDFD), and the rosiglitazone treated group (AAN + Rosi). After treating for 8 weeks, 24 h urine and blood samples were collected and the mice sacrificed to study the biochemical parameters associated with renal function. The samples were analyzed for renal fibrosis and mitochondrial dysfunction (MtD) markers. To achieve that, collagen III, collagen I, mitochondrial DNA copy numbers (mtDNA), mitochondrial membrane potential (MMP), ATP content, and ROS production were evaluated.* Results*. Our results showed that proteinuria, kidney function, and the renal pathological characteristics were improved by DFD and rosiglitazone. The expression of collagen III and collagen I decreased after treating with either DFD or rosiglitazone. Mitochondrial dysfunction based on the increase in ROS production, decrease in mitochondrial DNA copy numbers, and reduction of MMP and ATP content was improved by DFD and rosiglitazone.* Conclusions*. DFD could protect against renal impairments and ameliorate mitochondrial dysfunction in chronic AAN mice.

## 1. Introduction

Natural and alternative medicines are widely used to treat and prevent different kinds of diseases worldwide. However, these products, when prepared incorrectly, can result in nephrotoxicity. The patient may develop electrolyte abnormalities, proteinuria, acute kidney injury, and chronic kidney disease (CKD). Aristolochic acid nephropathy (AAN) is one of the best-known iatrogenic kidney diseases caused by* Aristolochia* herbal medications [1]. AAN is a rapidly progressive interstitial nephropathy that can lead to end-stage renal disease. Previous AAN studies have mostly focused on the aristolochic acids- (AA-) induced acute tubular necrosis and acute nephrotoxicity [2]. The pathological mechanisms responsible for chronic AAN are poorly understood.

Mitochondrial dysfunction usually involves an increase in production of mitochondria-derived ROS, breakdown of membrane potential, and abnormality of energy metabolism. It is usually caused by mitochondrial DNA mutations, resulting from the use of certain drugs and chemicals [3]. Previous studies have suggested that mitochondrial dysfunction (MtD) is associated with a variety of progressive kidney diseases, including focal segmental glomerular sclerosis (FSGS) [3, 4]. Other studies have suggested that mtDNA mutations resulting from A-to-G transition at the nucleotide 3243 in the tRNA^leu^ (UUR) gene and a 4696-bp deletion may be involved in the development of FSGS [4]. A growing number of studies have also shown that MtD plays an essential role in rodent FSGS models for puromycin aminonucleoside nephrosis (PAN) and aldosterone-induced renal injury [4, 5]. However, the role of MtD in chronic AAN has not been studied.

Dahuang Fuzi Decoction (DFD) is a traditional formula consisting of Da Huang (Radix et Rhizoma Rhei), Pao-fuZi (Radix Aconiti Lateralis Preparata), and Xi Xin (Radix et Rhizoma Asari) [6]. In clinics, DFD has been widely used to treat chronic dysentery, acute ileus, and CKD [7]. Most importantly, DFD has been shown to improve renal function significantly and the quality of life for CKD patients [6]. A high performance liquid chromatography-mass spectrometry (HPLC-MS) study found that three anthraquinones including rhein, aloe-emodin, and emodin are presented in rat plasma treated with DFD [7], suggesting that these compounds may improve renal function. However, the underlying mechanisms by which DFD may improve renal function are poorly understood.

In the present study, we established a suitable chronic AAN mice model with mice by administering intraperitoneal injections of aristolochic acids I (AA I) and subsequently found pronounced MtD in their kidney tissues. In addition, our results suggest that DFD protect against AA I-induced renal fibrosis and ameliorate MtD and oxidative stress.

## 2. Materials and Methods

### 2.1. Reagents

Aristolochic acid I (A5512), rosiglitazone (R2408), and 2′,7′-dichlorofluorescein (DCF) diacetate (DCFDA) (D6883) were obtained from Sigma. The primary antibodies used in this study were rabbit anti-collagen I, rabbit anti-collagen III, and rabbit anti-GAPDH (BS1530; BS1531; AP0063; Bioworld Technology).

### 2.2. Preparation of DFD

The granules of DFD included Radix et Rhizoma Rhei (9 g, voucher specimen number 1201080), Radix Aconiti Lateralis Preparata (12 g, voucher specimen number 1210003), and Radix et Rhizoma Asari (3 g, voucher specimen number 1208107), which were purchased from Tianjiang Pharmacology Co. Ltd. (Jiangyin, China). All these herb granules, with a total weight of 24 g, were dissolved in 24 mL saline at a concentration of 1 g/mL.

### 2.3. Animals and Drug Treatment

All experiments animals were approved by the Animal Ethics Committee of Nanjing University of Chinese Medicine. Male mice that were 8 weeks old, with a C57BL/6 background, were purchased from the Experimental Laboratory of Animal Models (Nanjing, China) and housed in the Experimental Animal Center of Nanjing University of Chinese Medicine. The mice were randomly divided into 6 groups of 6 mice per group. The AAN group received intraperitoneal injections of 3 mg/Kg AA I every 3 days for 8 weeks. Four weeks after the AA I treatment, a normal dose (2.5 g/kg/d) of DFD was administered by oral gavage to one group (NDFD). A high dose (5 g/kg/d) was also administered by oral gavage to another group (HDFD). The negative control mice (control) received injections and oral gavage of saline. The vehicle control mice (AAN + vehicle) received saline by oral gavage for 4 weeks after the AA I treatment. The rosiglitazone treated group (AAN + Rosi) received 5 mg/kg/d of Rosi by oral gavage for 4 weeks following the AA I treatment. Blood and kidney samples were collected for further examination at the end of 8-week treatment period, and the mice were sacrificed.

### 2.4. Renal Function Tests

Serum blood urea nitrogen (BUN), serum creatinine (Scr), and urinary protein (Upro) were measured by commercial kits according to the manufacturer's instructions.

### 2.5. Masson Trichrome Staining

The kidney samples were fixed with 4% paraformaldehyde, embedded in paraffin, and sectioned transversely. Kidney sections (2 *μ*m) were stained with Masson trichrome. The pathological changes were observed under a light microscope and photographs were obtained.

### 2.6. Western Blotting Analysis

To identify the expression profiles of collagen I and collagen III, lysates of cultured kidney tissues from different groups were collected and analyzed. The samples were washed with PBS and homogenized in cell lysis buffer containing a cocktail of protease inhibitors and phosphatase inhibitors. Sample lysates were separated by SDS-PAGE and electronically transferred onto polyvinylidene fluoride membranes. Membranes were blocked in 5% BSA for 1 h at room temperature and incubated overnight at 4°C with the anti-collagen I and anti-collagen III antibodies, respectively. After washing with TBST, membranes were incubated with corresponding HRP-conjugated secondary antibodies for 1 h at room temperature. The blots were visualized with the Amersham ECL Detection System (Amersham, Buckinghamshire, UK). Image J software was used to quantify and standardise band intensities.

### 2.7. Mitochondrial Membrane Potential (MMP)

Kidney tissues were dissociated into cells through tryptase digestion. The dissociated cells were incubated with JC-1 (7.5 *μ*M) in the dark for 30 min at 37°C. The stained cells were then washed with JC-1 washing buffer before subjected to flow cytometry analysis. Densitometry from MMP was calculated using the ratio of JC-1^+^ numbers to JC-1^−^ numbers.

### 2.8. Quantitative Real-Time PCR

Total DNA from renal cortex was isolated using a DNeasy Tissue Kit (Invitrogen). The target gene and reference gene were detected with real-time PCR. Amplification was performed using an ABI 7500 real-time PCR Detection System (Foster City, CA) with FastStart Universal SYBR Green master mix (Roche, Germany). Thermal cycling conditions were 95°C for 10 min followed by 40 cycles of 95°C for 15 s and 60°C for 1 min. Primer 3 software was used to design the primers (http://Frodo.wi.mit.edu). Relative amounts of mtDNA copy numbers were normalized to the nuclear 18S rRNA. The primer pair sequences are shown as follows: mtDNA, Forward: 5′TTTTATCTGCATCTGAGTTTAATCCTGT3′, Reverse: 5′CCACTTCATCTTACCATTTATTATCGC3′; 18SrRNA, Forward: 5′GGACCTGGAACTGGCAACAT3′, Reverse: 5′GCCCTGAACTCTTTTGTGAAG3′.

### 2.9. ATP Measurements

ATP levels in the renal cortex were detected using a luciferase-based bioluminescence assay kit following the manufacturer's instructions (Sigma). The luciferase activity was then measured using a FLUOstar Optima reader. Data are presented as the ATP levels normalized to protein concentrations.

### 2.10. Reactive Oxygen Species (ROS)

Intracellular ROS was assessed using the DCFDA kit (Sigma) as previously described [8]. Kidney cortex sections were labeled for ROS according to manufacturer's protocol. Samples were imaged using fluorescence microscope. Image J was used to quantify pixel density in glomerular. ROS generated by mitochondria were also measured with DCFDA kit.

### 2.11. Statistical Analysis

All data was analyzed using SPSS version 13.0 and shown as the mean ± SD. Student's* t*-test was used for comparisons between two groups, comparisons between multiple groups were analyzed by ANOVA, and a nonparametric test was used when necessary.* P *values of < 0.05 were considered statistically significant.

## 3. Results

### 3.1. DFD Ameliorated Disease Severity in the Chronic AAN Model

We investigated whether the DFD treatment could improve renal function in the chronic AAN model. The two DFD groups and the Rosi group were evaluated. Two physical and three nephropathy-related biochemical parameters were used to determine the disease severity in the chronic AAN model. As shown in Table 1, the AA I injection resulted in the loss of overall body weight (BW) and an increase in kidney weight/body weight (KW/BW) ratio compared to control mice. In addition, AA I caused a significant loss in the kidney function as characterized by elevated levels of blood urea nitrogen (BUN), serum creatinine (Scr), and urinary protein (Upro) compared to the control group. Similar results were seen in the vehicle treatment group. There was no improvement in renal function in the NDFD group (Table 1), while significant improvements in BW, KW/BW, and other kidney function parameters were seen in the HDFD group (Table 1). As expected, the significant improvement was also observed in the Rosi group (Table 1).

### 3.2. DFD Reduced Collagen Deposition in the Chronic AAN Model

**Figure 1:**
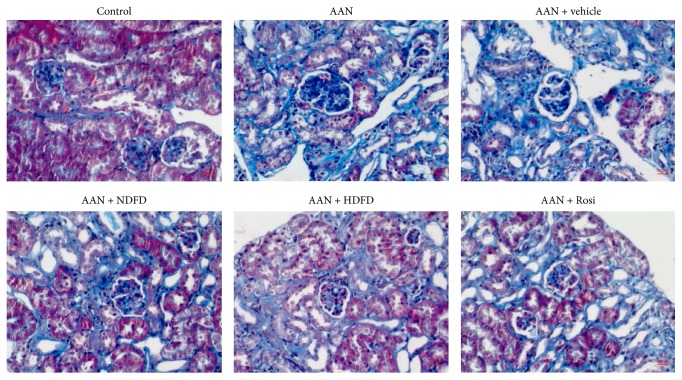
Effects of Dahuang Fuzi Decoction (DFD) on the formation of renal fibrosis in the renal tissues of chronic aristolochic acid nephropathy (AAN) mice (*n* = 6 mice/group). Representative Masson trichrome staining (400x) was used to analyze renal fibrosis in kidneys from paraformaldehyde-fixed kidney sections taken from each group of mice.

Renal histology revealed glomerular enlargement, glomerular sclerosis, tubular dilatation, and dark-stained cell infiltration in AAN kidneys (Figure 1). Both HDFD and Rosi significantly improved physical and biochemical parameters in the AAN mice, while the NDFD group showed less improvement (Figure 1). Furthermore, in the normal kidney tissue, blue stained collagen was only observed around the basement membranes and blood vessels (Figure 1). In the AAN and vehicle mice, excessive deposition of collagen was observed around the kidney tissue, suggesting an increase in the proliferation of fibroblasts and secretion of collagen (Figure 1). In addition, the HDFD and Rosi groups showed a significant reduction in collagen deposition. Conversely, in the NDFD group, the DFD treatment showed less reduction on collagen depositions (Figure 1).

**Figure 2:**
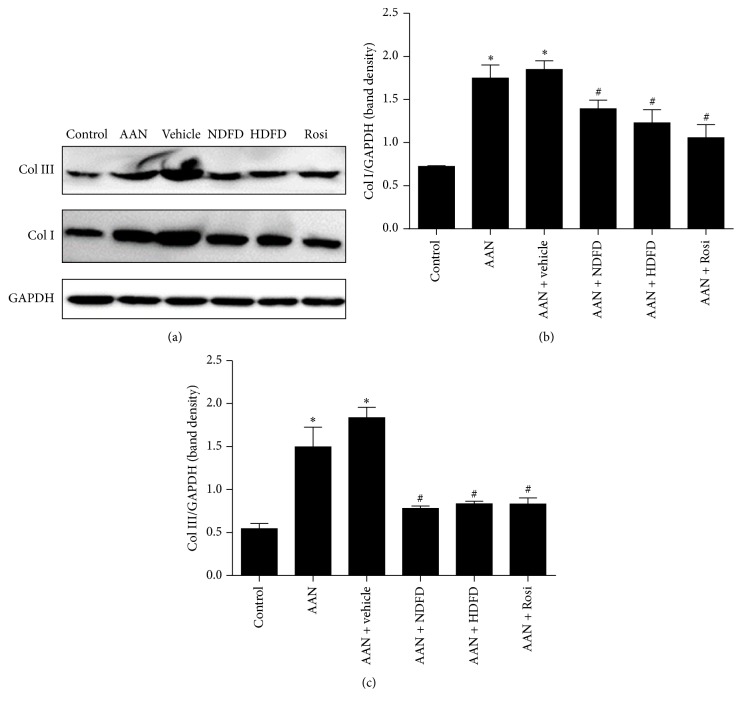
Western blot analysis for detecting the expressions of collagen I and collagen III in renal tissues. (a) Representative immunoblots obtained from control, AAN, AAN + vehicle, AAN + NDFD, AAN + HDFD, and AAN + Rosi are shown. (b, c) Densitometry analysis for Western blot results of collagen I and collagen III protein in extracts prepared from 6 mice in each group. ^*∗*^*P* < 0.05 versus control. ^#^*P* < 0.05 versus AAN.

In addition to histological examinations, a Western blot analysis was performed to confirm the overexpression of collagen I and collagen III in renal tissues. The results showed that, in the kidney tissue of the AAN and vehicle groups, protein levels of collagen I and collagen III were increased in comparison to the control mice (Figure 2(a)). The expression of collagen I and collagen III was inhibited in the NDFD, HDFD, and Rosi groups (Figure 2(a)). The quantitative analysis of protein expression was shown in Figures 2(b) and 2(c). This finding is consistent with the histologic examination above, suggesting that DFD could protect against AA I-induced nephropathy by reversing the overexpression of collagen.

### 3.3. DFD Ameliorated AA I-Induced MtD in Mice Kidneys

**Figure 3:**
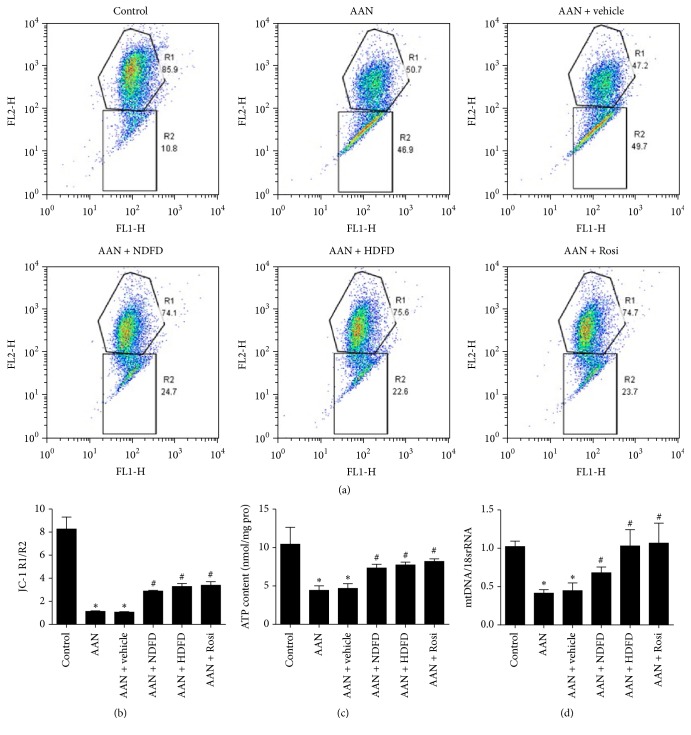
Effect of Dahuang Fuzi Decoction (DFD) on mitochondrial function in chronic aristolochic acid nephropathy (AAN) mice. (a) Mitochondrial membrane potential (MMP) analyzed by flow cytometry. (b) Densitometry analysis of MMP. (c) Adenosine-50-triphosphate (ATP) production. (d) Mitochondrial DNA (mtDNA) copy numbers. Data shown as mean ± SD (*n* = 6). ^*∗*^*P* < 0.05 versus control; ^#^*P* < 0.05 versus AAN.

Previous studies have shown that MtD is involved in progressive nephropathies. We studied whether DFD may improve mitochondrial function in the AAN model. To achieve that, mitochondrial membrane potential (MMP), ATP levels, and mtDNA copy numbers were analyzed. As expected, AA I injections caused a significant decrease of MMP in AAN mouse kidneys compared to controls (Figure 3(a)). This effect was significantly reduced by DFD and Rosi (Figure 3(a)). The densitometry analyses of MMP were shown in Figure 3(b). Consistent with MMP results, the reduction of ATP production and mtDNA copy numbers in AAN mice were significantly improved by DFD and Rosi (Figures 3(c) and 3(d)).

### 3.4. DFD Reduced AA I-Induced ROS in Mice Kidneys

**Figure 4:**
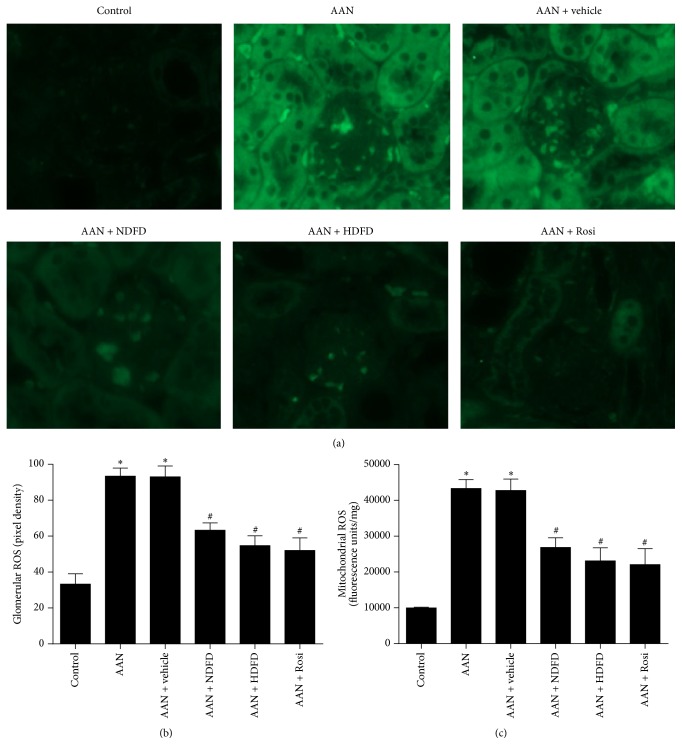
Effect of Dahuang Fuzi Decoction (DFD) on kidney ROS production in chronic aristolochic acid nephropathy (AAN) mice. (a) Detection of oxidative products using 2′,7′-dichlorofluorescein (DCF) to measure hydrogen peroxide in the kidney (×200). (b) Quantification of the pixel density of DCF staining in glomeruli. (c) Kidney mitochondrial levels of reactive oxygen species (ROS). Data are shown as mean ± SD (*n* = 6). ^*∗*^*P* < 0.01 versus control; ^#^*P* < 0.01 versus AAN mice by analysis of variance.

Accumulation of reactive oxygen species (ROS) is responsible for causing oxidative stress, which in turn can cause renal damage. We examined ROS production levels of glomerular and mitochondrial ROS in AAN kidneys. AA I-induced significant ROS production in mouse kidneys, both in the glomerulus and in the tubules (Figures 4(a) and 4(b)). This effect was significantly reduced by DFD and Rosi treatments (Figures 4(a) and 4(b)). Additionally, the mitochondrial swelling was significantly reduced by DFD and Rosi, as compared to AAN controls (Figures 4(a) and 4(b)). AAN mice showed a significant increase in mitochondrial ROS production (Figure 4(c)). Conversely, DFD and Rosi significantly reduced the levels of ROS in mitochondria (Figure 4(c)). These findings suggest that DFD protect against the renal damage and inhibit MtD induced by AA I.

## 4. Discussion

AAN is one of the most commonly iatrogenic kidney diseases caused by herbs rich in AA. Among the AA types, AA I is the main chemical component that causes nephrotoxicity [9]. The clinical presentation of AAN was not recognized initially as being caused by AA I. There were few cases reported of Fanconi syndrome and acute kidney injury due to tubular necrosis following exposure to AA I [10, 11]. Initial studies that focused on the chronic progress of AAN found that the rats with AAN exhibited remarkable azotemia and minimal proteinuria after 8 weeks of AA-induced nephrotoxicity. The histology of the kidney exhibited heavy inflammatory cell infiltration, tubular dilation, and interstitial fibrosis [2, 12]. In the present study, we used AA I to establish a chronic AAN model through intraperitoneal injections for 8 weeks. At the end of study, we found that the kidney function was significantly impaired as reflected by increased levels of BUN and Scr. Histological examinations showed tubular dilatation, dark-stained cell infiltration, and excessive deposition of collagen in the kidneys of the AAN group as demonstrated by Masson trichrome staining and western blotting. Based on these data we determined that the AAN model could be used to investigate the pathophysiological mechanisms responsible for chronic AAN.

Traditional Chinese medicine (TCM) has long been used to treat various kidney diseases in China. More importantly, it has been proven to be effective. DFD, which is frequently used in TCM, has been shown to be effective in promoting diuresis, reducing proteinuria, and restoring renal function in patients with CKD [6]. Using the chronic AAN model, we demonstrated that oral administration of DFD at the dose of 5g/kg/d significantly decreased the levels of BUN, serum creatinine, and proteinuria and improved the histologic characteristics of renal fibrosis. TCM treatment usually involves a combination of multiple herbs rather than a single compound. As previously reported, DFD contains at least three bioactive compounds, which include rhein, aloe-emodin, and emodin [7]. Previous studies have shown that rhein can attenuate the expression of *α*-smooth muscle actin (*α*-SMA) and the deposition of fibronectin (FN) in rat kidney, which in turn may improve kidney fibrosis [13, 14]. Additionally, emodin was found to be effective in suppressing NF-*κ*B-mediated TGF-*β*1 and FN overexpression, thereby protecting against diabetic nephropathy [15]. Aloe-emodin is a variant of emodin, which has similar effects as emodin, and has been found to reduce acute liver injury in rats [16]. Whether aloe-emodin is capable of improving renal function is unclear. It is possible that the three bioactive anthraquinones in DFD act synergistically to improve renal function in the chronic AAN model. Future studies are needed to investigate the role of the compounds present in DFD in treating chronic AAN and other kidney disease models.

The involvement of MtD in the pathogenesis of kidney diseases has been widely accepted, including CKD and acute kidney injury (AKI). Recent studies have reported that the peroxisome proliferator-activated receptor-*γ* coactivator (PGC)-1*α*-mitochondria axis is involved in MtD both in vitro and in vivo [17, 18]. The kidney is rich in mitochondria with a high potential of exhibiting significant MtD [19]. In kidney tissues, MtD may generate ROS, which may cause damage of cells and tissue and result in kidney disease [20, 21]. Studies have shown that MtD may be involved in the development of FSGS in humans and may play a role in puromycin and aldosterone-induced renal injury [3, 19]. In the present study, we reported that MtD was also present in AA I-induced renal injury, as characterized by decreased MMP and ATP production levels, reduced mtDNA copy numbers, and overproduction of ROS. Furthermore, our data showed that DFD and Rosi significantly reduced MtD in the kidney and subsequently ROS accumulation, which might contribute to the improvement of renal function in chronic AAN mice. However, the mechanisms by which the active compounds in DFD restore mitochondrial function in injured kidneys have not been determined.

## 5. Conclusion

The present study showed that DFD could protect AA I-induced renal fibrosis and improve mitochondrial dysfunction in mouse kidneys. We conclude that DFD could be a promising therapeutic agent for the treatment of chronic AAN and other kidney diseases caused by MtD.

## Figures and Tables

**Table 1:** Physical and biochemical parameters in control and treatment groups.

	Control	AAN	AAN + vehicle	AAN + NDFD	AAN + HDFD	AAN + Rosi
BW (g)	28.30 ± 1.28	20.61 ± 1.22^*∗*^	20.22 ± 1.28^*∗*^	22.71 ± 1.38	23.94 ± 1.51^#^	24.61 ± 2.1^#^
KW/BW (mg/g)	10.32 ± 0.16	14.32 ± 0.76^*∗*^	13.92 ± 0.81^*∗*^	11.82 ± 0.22^#^	11.31 ± 0.16^#^	11.02 ± 0.46^#^
BUN (mmol/L)	5.78 ± 0.17	7.98 ± 0.67^*∗*^	7.65 ± 0.56^*∗*^	6.78 ± 0.37	6.38 ± 0.46^#^	6.45 ± 0.27^#^
Scr (*μ*mol/L)	15.15 ± 1.26	19.95 ± 2.51^*∗*^	18.95 ± 2.76^*∗*^	17.15 ± 1.06	16.25 ± 1.07^#^	16.11 ± 1.22^#^
Upro (mg/24 h)	0.51 ± 0.23	2.75 ± 1.12^*∗*^	2.81 ± 0.92^*∗*^	1.32 ± 0.42	0.98 ± 0.34^#^	0.87 ± 0.36^#^

Body weight (BW); kidney weight (KW); urinary protein (Upro); serum blood urea nitrogen (BUN); serum creatinine (Scr); data shown as mean ± SD. ^*∗*^*P* < 0.05 versus control. ^#^*P* < 0.05 versus AAN.
